# Artificial intelligence in nursing: A journey from data to wisdom

**DOI:** 10.1097/NSG.0000000000000165

**Published:** 2025-03-24

**Authors:** Kathleen McGrow

**Affiliations:** **Kathleen McGrow** is the Global Chief Nursing Innovation Officer at Microsoft Health & Life Sciences in Redmond, Wash., and an adjunct clinical instructor at The University of Alabama at Birmingham School of Nursing.

**Keywords:** AI, artificial intelligence, Data, Information, Knowledge, Wisdom, Framework, DIKW, data quality, machine learning, nursing

## Abstract

Artificial intelligence (AI) can enhance nursing practice by assisting in clinical decisions, patient outcomes, and operational efficiencies. This article explores the role of AI in decision-making, data management, and task automation within the Data, Information, Knowledge, Wisdom Framework. It also addresses data quality, ethical considerations, and the need for continuous AI system improvement, emphasizing AI as a valuable healthcare partner.

## Abstract 2

This article explores the role of artificial intelligence (AI) in decision-making, data management, and task automation within the Data, Information, Knowledge, Wisdom Framework. It also addresses data quality, ethical considerations, and the need for continuous AI system improvement.

## Artificial intelligence in healthcare

Artificial intelligence (AI) is a transformative force in the healthcare industry, providing information technology (IT) systems with various capabilities similar to human cognitive functions. The term AI broadly encapsulates machine-based capabilities that demonstrate cognitive skills comparable to those of humans.^1^ AI adds value by automating routine tasks and enhancing human decision-making with forecasts and insights gleaned from extensive datasets.

**Figure:**
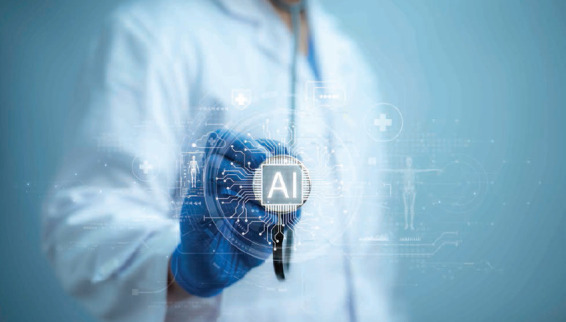
No caption available.

Integrating AI into nursing practice holds significant promise. As nurses manage AI-driven analytics and patient-care technologies, their role in healthcare becomes even more crucial. AI supports decision-making by analyzing vast datasets and providing insights that enhance clinical judgment and improve patient outcomes.^2^ AI can streamline data management, enabling nurses to focus more on patient care by ensuring accurate and timely information. This technology can be used to personalize patient care through machine learning algorithms, tailoring interventions based on individual characteristics to optimize treatment plans and enhance satisfaction. Additionally, AI automates routine tasks like documentation and administrative duties, giving nurses more time for direct patient care.^2^

As nurses embrace AI, they become pivotal in leveraging technology to improve patient outcomes, operational efficiency, and healthcare delivery. The journey of integrating AI into nursing practice promises exciting advancements and better patient experiences. AI complements nurses' expertise; it does not replace critical thinking or empathy but augments decision-making. As AI matures, nurses can harness its capabilities to improve patient outcomes and streamline clinical workflows.

## Data, Information, Knowledge, Wisdom (DIKW) Framework

The Data-Information-Knowledge-Wisdom (DIKW) Framework provides a structured approach to understanding and utilizing data effectively.^3^,^4^ The use of Health Information Technology (HIT), which includes the practice of storing, sharing, and analyzing healthcare data, along with the DIKW Framework could enhance nurses' clinical reasoning, contributing to better patient outcomes (see *DIKW Framework*).^3^,^5^

The framework starts with raw data, such as patient vital signs and bloodwork results, which are unprocessed and lack context. When these data are processed (analyzed and organized) and contextualized (interpreted within a relevant framework), they become information, such as trends or potential drug interactions. Knowledge is created by synthesizing this information into actionable insights, enabling nurses to make informed decisions, such as recognizing early signs of sepsis or an adverse drug reaction.

Wisdom goes beyond knowledge, allowing experienced nurses to make holistic judgments and prioritize care. Experienced nurses draw upon their accumulated knowledge, intuition, and empathy to assess complex situations, consider the broader context of a patient's needs, and make informed decisions beyond standard protocols. This dynamic process of integrating knowledge with practical experience and compassionate understanding is what truly defines wisdom in nursing.

**Figure:**
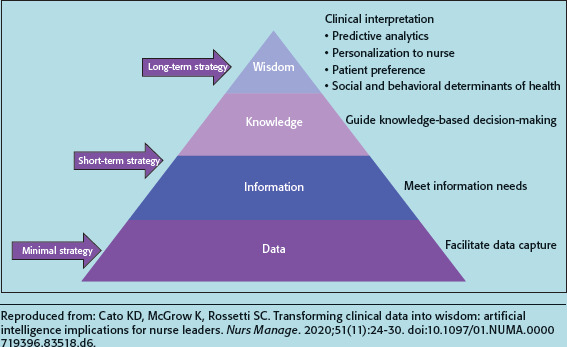
DIKW Framework

## Applying the DIKW Framework in nursing practice

The DIKW Framework is a roadmap for nurses to navigate the complexities of data utilization in clinical practice.^5^ AI enhances each stage of the DIKW Framework. At the *data level*, AI can help collect large volumes of patient data efficiently and accurately. For instance, natural language processing (NLP) algorithms leveraging AI can extract relevant information from clinical notes, reducing manual electronic health record (EHR) review time. AI-driven tools can automate data entry, minimizing the risk of errors and freeing nurses to focus on patient care.

At the *information level*, AI algorithms identify patterns or trends within complex datasets. For nurses, this means recognizing subtle changes in vital signs, lab results, or medication adherence. AI-powered predictive models can alert nurses to deteriorating patient conditions, allowing timely interventions.

At the *knowledge level*, AI systems can integrate information from various sources, such as the EHR and research articles, to support clinical decision-making. For instance, AI can recommend evidence-based interventions based on patient-specific data. AI-driven decision support tools provide real-time guidance, suggesting appropriate treatments, dosage adjustments, or potential adverse reactions.

At the *wisdom level*, advanced AI, such as deep learning (DL) algorithms, can analyze complex ethical dilemmas. For instance, AI can assist in allocating scarce resources during a pandemic or predicting patient outcomes based on personalized risk factors.^5^ Although AI contributes to wisdom, it cannot replace human intuition, empathy, or the ability to consider the patient's context. Nurses remain essential in interpreting AI-generated insights and making compassionate, patient-centered decisions.

AI is a valuable partner in the DIKW journey, but its successful integration relies on nurses' expertise and ethical judgment. By embracing AI while maintaining a patient-centric approach, nurses can harness the full potential of data-driven healthcare.

## Potential impact on clinical practice

AI analyzes vast amounts of data, extracting patterns and insights that aid clinical decision-making. For instance, predictive models can identify patients at risk for deterioration, allowing timely interventions.^5^ AI also assists in diagnosing diseases by analyzing medical images such as X-rays and computed tomography scans. DL algorithms can detect subtle abnormalities, supporting accurate diagnoses. However, as AI becomes more integrated, nurses must navigate ethical dilemmas, such as balancing AI recommendations with patient preferences and individual contexts.

## Machine learning (ML)

ML is a subset of AI focusing on algorithms that learn from data to make predictions and decisions without explicit programming for each task. ML streamlines routine activities, such as clinical administrative processes.^6^ Automating repetitive administrative tasks such as inventory management or appointment scheduling give nurses more time for direct patient care. ML analyzes vast amounts of data, extracting patterns and insights.^5^ For nurses, predictive models can identify patients at risk for deterioration, such as sepsis risk models^5^ and modified early warning systems (MEWS) to enable timely interventions. ML empowers nurses by automating tasks, enhancing decision-making, and uncovering insights from healthcare data. Ethical factors and human insights are crucial to the effective integration of ML.

## Predictive analytics

Predictive analytics is a subset of AI that employs data, statistical algorithms, and ML techniques to identify the likelihood of future outcomes.^7^ Predictive models analyze historical patient data, identifying patterns and trends. These models can predict readmissions, complications, or disease progression. By leveraging predictive analytics, nurses gain insights into individual patient risks, allowing proactive interventions.

Predictive analytics assists in tailoring treatment plans. For instance, it can recommend personalized medication dosages based on patient characteristics and response patterns. Predictive analytics empower nurses by providing foresight, enabling targeted interventions, and enhancing patient care and resource allocation, thereby improving patient outcomes. Ethical considerations and human expertise remain essential for successfully implementing predictive models.

## Natural language processing (NLP)

NLP is a subset of AI that focuses on enabling computers to understand, interpret, and generate human language.^2^ NLP algorithms can analyze clinical notes, discharge summaries, and other textual data. By extracting relevant information, NLP can help clinicians quickly grasp patient histories, diagnoses, and treatment plans. For instance, NLP can identify critical terms like medications, procedures, or symptoms; streamline EHR review; and ensure accurate data capture (see *NLP examples for nurses*). NLP automates tasks such as coding diagnoses, extracting lab results, and identifying adverse events. This reduces the administrative burden, allowing nurses to focus on direct patient care. By converting unstructured text into structured data, NLP facilitates data integration across EHRs.

NLP presents several legal challenges that require careful consideration. One of the primary concerns is data privacy and security, particularly the handling of Protected Health Information (PHI). NLP systems must comply with regulations such as the Health Insurance Portability and Accountability Act (HIPAA)^8^ in the US, ensuring that patient data are processed securely to maintain confidentiality and prevent data breaches. Additionally, obtaining informed consent from patients about how their data will be used, including for NLP applications, is crucial to meet legal standards.

NLP-powered clinical decision support systems analyze vast volumes of literature, guidelines, and research articles. Nurses can benefit from NLP-generated evidence-based insights, such as alerts for potential drug interactions and personalized care plans based on patient-specific information. NLP empowers nurses by transforming text into actionable insights. While embracing NLP, nurses remain essential in interpreting context and ensuring patient-centered care.

## DL

DL is a subset of ML based on neural networks with representation learning. It uses complex multilayer neural networks to process data using algorithms that can learn from data and gain new insights.^9^ DL algorithms constitute the foundation of the most sophisticated AI models.

### Generative AI

Generative AI, an exciting advancement within DL, has the remarkable ability to create new content by recognizing patterns from existing data.^10^ Large language models (LLMs) and large multimodal models (LMMs) leverage generative AI techniques to create new content. LLMs and LMMs, such as Generative Pretrained Transformer 3 (GPT-3) and its successors, play a pivotal role in generative AI.^10^ These models are trained on extensive text data, learning to predict the next word in a sentence.^9^ For nurses, LLMs offer several applications. LLMs can assist in generating patient notes, summaries, and reports. LLMs can process medical jargon and context, making documentation more efficient. They can also generate patient-friendly materials explaining diagnoses, treatments, and medications. Nurses may use these resources to enhance patient education of their disease and treatment plans. LLMs summarize medical literature, aiding nurses in staying up to date with evidence-based practices.

LMMs take generative AI further by incorporating multiple modalities, such as text, video, images, and sound.^10^ Healthcare is inherently multimodal, involving diverse data types such as text, images, audio, and videos. LMMs can assist in processing context by handling text, visual, and auditory cues. For instance, imagine a model that reads patient notes, analyzes X-rays, and simultaneously processes recorded symptoms. For nurses, this means enhanced context awareness (see *LLM example for nurses*).

**Figure:**
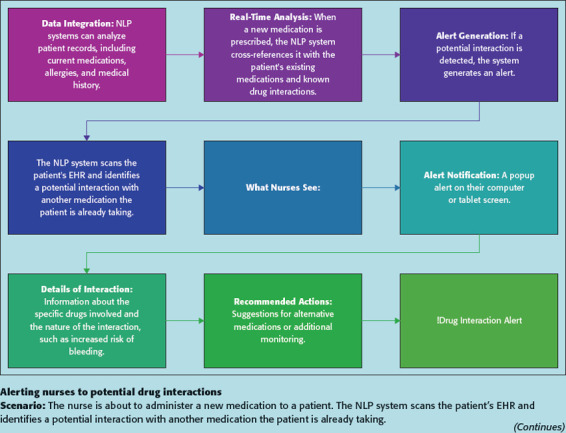
NLP examples for nurses

**Figure:**
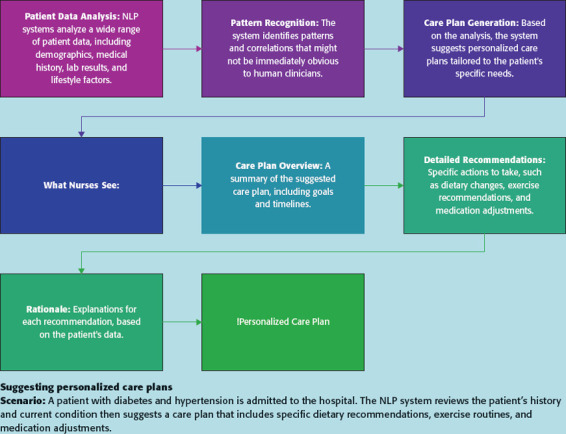
NLP examples for nurses *(Continued)*

LMMs can analyze ECGs alongside clinical reports, providing a holistic view of patient health. Beyond text responses, LMMs can generate comprehensive documents, such as discharge summaries, incorporating images, lab results, and treatment plans.

Synthetic data are artificially generated and nonreversible, designed to mirror statistical properties and relationships found in real-world data.^11^ In this context, nonreversible means that synthetic data cannot be traced back to or used to reconstruct the original real-world data from which it was derived. Unlike deidentified data, synthetic data are created from scratch rather than derived from individual patient records, ensuring it cannot be deanonymized and providing an additional layer of privacy while preserving the original data's value.^11^ Synthetic data must be handled and stored in secure environments that guarantee privacy and data integrity. This usually means employing encrypted storage solutions and secure cloud services that adhere to healthcare regulations. Nurses can access diverse data for evidence-based practice while safeguarding patient confidentiality.

For instance, LLMs can generate realistic ECG waveforms for algorithm training. ECG waveforms are an example of using synthetic data to train ML models so that algorithms can be developed to learn more about ECGs. These synthetic waveforms could be used for Advanced Cardiovascular Life Support training.

## Challenges and best practices

### Data quality

Successful AI implementation in healthcare is deeply connected to the quality of the data in these systems' processes. Data quality directly influences AI models' performance, accuracy, and reliability.^12^ AI models can make better predictions and produce more reliable outcomes when they have access to high-quality data. This, in turn, fosters trust and confidence among users, including nurses. Data quality is crucial for nurses who depend on data to inform patient care and make treatment choices. The goal of data quality management in healthcare organizations today is to ensure that the data meet the organization's business processes, decision-making, and planning needs.

On the other hand, poor data quality can lead to flawed results, causing the AI system to perform poorly or even fail. Therefore, data quality must be ensured. This entails identifying and preventing any biases in the data to make sure they are not perpetuated or amplified in the AI-generated output.^12^ Moreover, a diverse and representative dataset enhances an AI model's ability to generalize well across different situations and inputs. This ensures the model's performance and relevance across various contexts and user groups, including nurses. Therefore, ensuring data quality is not just a technical requirement but also a strategic one.^13^

Data integration plays a pivotal role in healthcare, especially for nurses at the forefront of patient care and data management. Data integration is merging data from diverse sources and presenting it in a unified format for comprehensive analysis, reporting, and decision-making.^14^ This process is indispensable for healthcare organizations that aim to amalgamate data from various systems, applications, and databases to derive insights and support evidence-based decision-making.

AI and ML are integral to data integration, significantly enhancing the process by automating and streamlining various tasks. Implementing AI-powered data integration can lead to improved decision-making, enhanced operational efficiency, and a more agile response to changing business conditions.

Change management is a crucial element when introducing AI into healthcare settings. The AI Maturity Roadmap^15^ offers a comprehensive framework for effective and sustainable AI deployment in healthcare. The AI Maturity strategic plan highlights six primary areas of attention: organizational culture, governance structure, commercial implementation, value creation, maintenance and operational management, and data architecture.^15^ Within these domains lie specific themes and five stages of maturity, which span from “awareness” to “transformational.”^15^ The adoption of AI in healthcare systems reflects different levels of preparedness, requiring tailored approaches for varying maturity levels.

Effective change management frameworks assist leaders in setting clear objectives and fostering productive discussions for widespread agreement and commitment to AI implementation. The integration of AI in healthcare presents challenges such as regulatory issues, reimbursement, return on investments, integration with data sources, education, fit with clinical workflows, and ethical considerations.^2^ Therefore, effective change management is vital for successfully adopting AI in healthcare.

### Evaluating AI

The evaluation and continuous improvement of AI systems are essential for their success. This involves regularly assessing their performance, identifying areas for improvement, and making necessary adjustments. Continuous improvement ensures that AI systems remain effective and relevant over time. It also aids in identifying and mitigating potential risks and issues early on, thereby enhancing their reliability and effectiveness.

Education is critical to addressing the challenges in implementing AI systems in healthcare settings. In response to the need for further education on AI, a group of nurse informaticist thought leaders developed the “*5 Rights of AI in Healthcare”* as a resource for clinicians and healthcare leaders to critically evaluate AI-driven technologies (see *5 Rights of AI in Healthcare***)**.^16^ The “*5 Rights of AI in Healthcare*” concept draws inspiration from the “Five Rights of Medication Administration,” which originated in early 20th-century nursing practices.^16^ These rights guided nurses in administering medication safely and accurately to enhance patient safety and improve quality.

The “*5 Rights of AI in Healthcare*” is a practical framework that outlines a standard approach for AI application in healthcare settings, including understanding the problem and population, incorporating correct solutions for workflow, competency of merging analytical intelligence with clinical insight, using accurate data grounded in logical reasoning, and ensuring essential protections. Using AI responsibly requires ongoing discussion, involving stakeholders, and regular review to guarantee effective deployment and uptake.^16^,^17^

**Figure:**
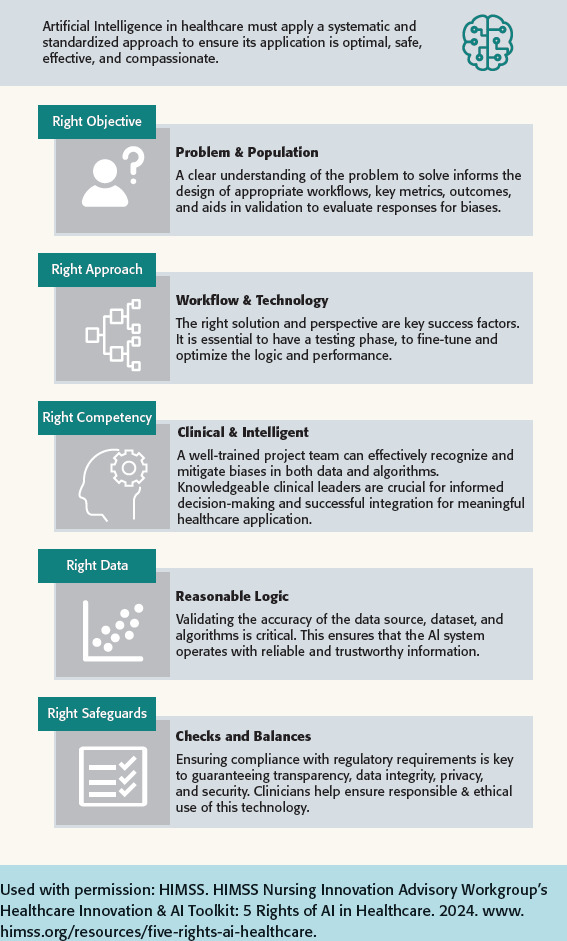
5 Rights of AI in Healthcare

The World Health Organization^13^ emphasizes the necessity of adhering to ethical principles and proper governance, as outlined in their guidance on the ethics and governance of AI for health, during the design, development, and deployment of AI in healthcare.^18^ The White House Blueprint for an AI Bill of Rights has proposed essential AI principles, including safe and effective systems, algorithmic discrimination protections to prevent bias and ensure fairness, data privacy, notice and explanation, and human alternatives, consideration, and fallback, which are crucial for ensuring ethical and equitable AI deployment in healthcare.^19^ By integrating these ethical guidelines and educational frameworks, healthcare professionals can effectively navigate the complexities of AI implementation, ensuring that these technologies are used responsibly and to the greatest benefit of patient care.

The Translational Evaluation of Healthcare AI framework suggests a comprehensive evaluation approach with three main components: capability assessment, utility evaluation, and adoption consideration.^20^ Capability assessment involves a detailed analysis of the technical competencies of AI systems, ensuring they fulfill their intended roles while maintaining data integrity and internal and external validity. It also includes meeting established performance benchmarks. The component of utility evaluation examines the practicality of AI systems, focusing on their broad applicability, safety measures, quality standards, transparency, privacy concerns, and adherence to the principle of no harm. This ensures that AI systems serve a beneficial and ethical role in healthcare settings. The adoption consideration addresses the practical implementation of AI systems within healthcare environments. It emphasizes the application, seamless technologic integration, service extent, and alignment with healthcare domains to facilitate widespread acceptance and operational efficiency.^20^

These approaches and frameworks aim to guarantee that AI technologies are technically robust, ethically aligned, and functionally beneficial within healthcare. Consequently, ongoing assessment and consistent enhancement are fundamental practices for the effective deployment of AI.

## Future directions and opportunities

There are exciting opportunities for nurses in the realm of AI. Nurses are positioned to collaborate closely with technology developers to align AI solutions with the realities of nursing workflows and critical clinical requirements. Nurses' direct perspectives and expertise are crucial in crafting AI resources that are functional and valuable within the everyday context of healthcare.

Nurses play an essential role in overseeing and protecting healthcare data. They can collaborate with data experts and HIT professionals to oversee the creation of AI algorithms that utilize robust datasets representing diverse populations. Nurses are integral in addressing the ethical dimensions of AI, including privacy, informed consent, inherent biases, and the openness of systems. Nurses can be advocates for ensuring AI implementations honor the integrity of patient care, uphold individual rights, and promote treatment equality.

Furthermore, nurses' involvement in the research and innovation of AI in healthcare is crucial. Leveraging their hands-on clinical understanding and patient management skills, nurses can bring critical insights into the development of AI utilities that genuinely enhance patient outcomes. Nurses should pursue ongoing education to remain at the forefront of AI advancements and their implications for healthcare. Staying current on emerging AI technologies empowers nurses to integrate these tools effectively into their caregiving and share knowledge with colleagues.

Nurse informaticists can utilize AI to enhance clinical workflows and patient outcomes. They can work with AI technologies to streamline processes, making them more efficient and effective. Nurse informaticists serve as a crucial bridge between healthcare professionals and technology creators in this context. They can ensure AI applications align with nursing processes and meet essential clinical conditions and workflows. Nurse informaticists can collaborate with data scientists and IT experts to establish a robust data framework. This framework ensures that AI algorithms are developed using reliable and inclusive datasets. Nurse informaticists can ensure AI systems prioritize patient safety, autonomy, and equitable care.

Although AI presents numerous opportunities, it is not meant to replace human judgment but to assist it. The critical thinking skills, empathy, and holistic care nurses provide are irreplaceable and will always be at the heart of nursing practice. AI tools are assistive technologies and not meant to replace human judgment, which is critical to ensure accuracy, safety, ethical standards, equity, and fairness.

## Conclusion

AI has become increasingly important in healthcare, particularly in nursing. It can automate routine tasks, provide decision-making support, facilitate personalized patient care, and streamline data management, thereby significantly enhancing the efficiency and effectiveness of healthcare delivery. Incorporating AI into the DIKW Framework has further empowered nursing professionals to leverage the full potential of data-driven healthcare. Extensive research is required to generate quantifiable evidence.

However, the successful implementation and integration of AI in healthcare presents many challenges, such as data quality, change management, and continuous evaluation. By addressing these challenges and adhering to best practices, healthcare organizations can harness the full potential of AI to enhance patient care and treatment decisions, particularly for nurses at the forefront of patient care and data management. This will lead to improved decision-making, enhanced operational efficiency, and a more agile response to changing business conditions.

Finally, although AI can augment human capabilities, it cannot supplant the intuition, empathy, and ethical judgment inherent in nursing practice. Remembering that AI is a tool designed to assist, not replace, human expertise is imperative. Nurses, therefore, remain at the epicenter of healthcare delivery, guiding the ethical use of AI and ensuring patient-centered care. The future of AI in healthcare holds exciting possibilities for nurses, promising to revolutionize healthcare delivery and improve patient outcomes. Nonetheless, the human touch in healthcare remains irreplaceable, underscoring the importance of maintaining a balanced, patient-centric approach in the era of AI.

## LLM example for nurses

Scenario: A nurse is caring for a patient with diabetes who has been admitted for hyperglycemia.

- Text Data: The LMM reads the patient's health history, including previous blood glucose levels and medication records.
- Visual Data: The LMM analyzes a recent photo of the patient's feet to check for any signs of diabetic foot ulcers.
- Audio Data: The LMM listens to a recording of the patient's description of their symptoms, such as feeling dizzy or having blurred vision.

By combining these data types, the LMM can provide the nurse with a clear picture of the patient's condition. It alerts the nurse to potential complications, like the risk of infection from a foot ulcer, and suggests actions, such as scheduling a consultation with a specialist. This helps the nurse make quick, informed decisions, ensuring the patient receives the best possible care.
